# Dimorphism and Dissemination of *Histoplasma capsulatum* in the Upper Respiratory Tract after Intranasal Infection of Bats and Mice with Mycelial Propagules

**DOI:** 10.4269/ajtmh.18-0788

**Published:** 2019-07-08

**Authors:** Roberto O. Suárez-Álvarez, Jorge H. Sahaza, Miriam Berzunza-Cruz, Ingeborg Becker, Everardo Curiel-Quesada, Armando Pérez-Torres, María del Rocío Reyes-Montes, Maria Lucia Taylor

**Affiliations:** 1 Unidad de Micología, Departamento de Microbiología-Parasitología, Facultad de Medicina, Universidad Nacional Autónoma de México, Ciudad de México, Mexico;; 2 Departamento de Micología, Instituto Nacional de Enfermedades Infecciosas- Administración Nacional de Laboratorios e Institutos de Salud “Dr. Carlos G. Malbrán”, Buenos Aires, Argentina;; 3 Unidad de Micología Médica y Experimental, Corporación para Investigaciones Biológicas, Medellín, Colombia;; 4 Departamento de Medicina Experimental, Facultad de Medicina, Universidad Nacional Autónoma de México, Ciudad de México, Mexico;; 5 Departamento de Bioquímica, Escuela Nacional de Ciencias Biológicas, Instituto Politécnico Nacional, Ciudad de México, Mexico;; 6 Departamento de Biología Celular y Tisular, Facultad de Medicina, Universidad Nacional Autónoma de México, Ciudad de México, Mexico

## Abstract

This article describes, for the first time, the role of the nasal mucosa (NM) as the initial site for the *Histoplasma capsulatum* mycelial-to-yeast transition. The results highlight that yeasts may arrive to the cervical lymph nodes (CLN) via phagocytes. Bats and mice were intranasally infected with *H. capsulatum* mycelial propagules and they were killed 10, 20, and 40 minutes and 1, 2, and 3 hours after infection. The NM and the CLN were monitored for fungal presence*.* Yeasts compatible with *H. capsulatum* were detected within the NM and the CLN dendritic cells (DCs) 2–3 hours postinfection, using immunohistochemistry. *Histoplasma capsulatum* was re-isolated by culturing at 28°C from the CLN of both mammalian hosts 2–3 hours postinfection. Reverse transcription-polymerase chain reaction assays were designed to identify fungal dimorphism, using mycelial-specific (*MS8*) and yeast-specific (*YPS3*) gene expression. This strategy supported fast fungal dimorphism in vivo, which began in the NM 1 hour postinfection (a time point when *MS8* and *YPS3* genes were expressed) and it was completed at 3 hours (a time point when only the *YPS3* transcripts were detected) in both bats and mice. The presence of intracellular yeasts in the nasal-associated lymphoid tissue (NALT), in the NM nonassociated with the NALT, and within the interdigitating DCs of the CLN suggests early fungal dissemination via the lymph vessels.

## INTRODUCTION

*Histoplasma capsulatum* is a globally widespread dimorphic fungus. This pathogen is the etiologic agent of histoplasmosis, a respiratory and systemic mycosis with different clinical presentations, depending on the host’s susceptibility, fungal virulence, and other factors associated with the infection process.^1^

This fungus grows in particular environments as a saprobe mycelial (M)-phase and produces infective propagules, mainly microconidia and small hyphal fragments. Once aerosolized and inhaled by hosts, these propagules can cause infection associated with a mild to severe clinical course, mostly in immunosuppressed individuals or in immunocompetent individuals exposed to high risks of infection in heavily contaminated places.

After inhalation, the infective M-phase propagules are thought to accumulate in the lung alveoli and to convert to the *H. capsulatum* parasitic and virulent yeast (Y)-phase within the alveolar macrophages. Usually, immunocompetent hosts can resolve the infection; however, under some circumstances, this pathogen can multiply in the hostile intracellular macrophage microenvironment, allowing the fungus to persist within the alveolar macrophages, thus leading to macrophage destruction and subsequent dissemination of its Y-phase.^2–4^

*Histoplasma capsulatum* develops in macrophages, neutrophils, and epithelial and dendritic cells (DCs) from different hosts.^2–6^ Processing and presenting *H. capsulatum* antigens to T-lymphocytes are generally more effective in DCs than in macrophages and these steps are necessary for triggering the adaptive immune response.^3,5^

A resident DC subpopulation in the lungs with a particular phenotype (CD11c^+^, F4/80^−^, CD11b^+^, Ly-6C^+^, and CD205^+^) was described to be responsible for the phagocytosis of *H. capsulatum* yeasts in C57BL/6 mice.^7^ Van Prooyen et al.^8^ also described the involvement of a specific DC subset (CD103+) in the lungs of female mice intranasally infected with *H. capsulatum* yeasts, identifying it as the primary cause for the type-I interferon (IFN-I) production in the infected host. They found that the toll-like receptors 7 and 9 (TLR7 and TLR9) were required for the IFN-I response, which restricted the fungal growth and favored the host survival.

Newman et al.^9^ found that the transition from conidia (M-phase) to yeasts (Y-phase) was restricted either in human or murine cultured lung DCs; however, in vivo infections using natural conditions in susceptible hosts can involve different steps at the onset of the interaction between *H. capsulatum* and the host’s tissues. Thus, because *H. capsulatum* conidia and hyphal fragments infect the host through the upper respiratory airway, the nose with the nasal mucosa (NM), including the nasal-associated lymphoid tissue (NALT) and the cervical lymph nodes (CLN), may be engaged during the first steps of the fungal morphotype’s transition, capture, and dissemination, which likely occur through the constitutive DCs, located strategically in the NM and the CLN.^1^ This study aimed at determining the roles played by the NALT, the NM, and the CLN as the initial anatomic sites for *H. capsulatum* M-to-Y morphotype transition and primary dissemination during the early stages of *H. capsulatum* intranasal infection of two different mammalian hosts. Pioneering strategies were implemented using bats (natural wild hosts) and mice (laboratory hosts) as experimental models to identify each fungal morphotype in the host´s infected tissues. To achieve this goal, we considered the expression of two morphotype-specific *H. capsulatum* genes: *MS8* (mycelial specific) expressed only in the fungal M-phase^10,11^ and *YPS3* (yeast specific) expressed only in the Y-phase.^12^ Thus far, this is most assuredly the only study that has used bats as experimental models to detect *H. capsulatum* dimorphism and dissemination.

## MATERIALS AND METHODS

### *Histoplasma capsulatum*.

The EH-53 strain, which was isolated from a patient infected in a cave in the state of Hidalgo, Mexico, was selected for the study. This strain is a reference from our laboratory, and it was characterized as an LAm A phylogenetic species by Kasuga et al.^13^ and confirmed by Vite-Garín et al.^14^ Recently, it was reclassified as an LAm A2 by Teixeira et al.^15^ It is deposited in the *H. capsulatum* Culture Collection of the Fungal Immunology Laboratory of the Department of Microbiology-Parasitology, in the School of Medicine, Universidad Nacional Autónoma de México (UNAM) (www.histoplas-mex.unam.mx), which is registered in the database of the World Data Centre for Microorganisms (WDCM) with number LIH-UNAM WDCM817.

### Animals.

Bats were used as infection models because of their possible evolutionary history with the pathogen *H. capsulatum* over millions of years in shared natural habitats,^16^ whereas mice used in the laboratory assays do not show this characteristic. Adult male *Tadarida brasiliensis* bats were captured in “El Salitre” cave, in the municipality of Meztitlán, state of Hidalgo, Mexico, with a chiropteran mist net. This insectivorous bat species was chosen because of its high susceptibility to *H. capsulatum* infection, because it is not in danger of extinction, and because bats from this species form large colonies in their shelters, facilitating their capture.^16^ Bats were transported alive from their shelters to the Fungal Immunology Laboratory, UNAM, where they were housed and maintained during a short time with coleopteran larvae and acidified water, until required for the assays. Eight-week-old male BALB/c mice were maintained at the animal facilities of the School of Medicine, UNAM, and they were provided with Rodent Laboratory Chow (PMI Nutrition International, LLC, Brentwood, MO) and water ad libitum. Both animal models were treated in accordance with the guidelines of the Ethics Committee of the School of Medicine, UNAM, in compliance with project numbers 112-2009 (approved on January 5, 2010) and 049-2011 (approved on June 14, 2011), and following the recommendations of the Animal Care and Use Committee of UNAM and the Mexican Official Guide (NOM 062-ZOO-1999).

### Fungal inoculum.

The M-phase of the EH-53 strain was cultured at 28°C in mycobiotic agar (Bioxon; Becton-Dickinson, Ciudad de México, Mexico), to be used as the fungal inoculum prepared according to Sahaza et al.^17^ with minor modifications. Cultures on mycobiotic agar were transferred to brain–heart infusion (BHI) agar (Bioxon) and incubated at 28°C for 4 weeks. After incubation, conidia and hyphae were harvested by gently washing the fungal culture surface with sterile saline solution (SS). This fungal suspension was filtered through Whatman filter paper (grade 40) to homogenize the inoculum and then centrifuged at 800 × *g* for 15 minutes at room temperature. The pellet was suspended in 1 mL of SS and observed under light microscopy to confirm the presence of a large number of microconidia and small hyphal fragments. The mycelial propagules’ viability was tested with trypan blue (0.05%), and to confirm viability, a sample was cultured in BHI agar at 28°C. The propagule suspension was carefully prepared in SS and adjusted to 0.5 optical density units, and the desired inoculum was quantified using a hemocytometer. The fungal inoculum (8 × 10^7^ mycelial propagules/100 μL) was standardized as homogeneously as possible, containing approximately 90% microconidia and 10% hyphal fragments. All procedures were performed under strict biosafety and sterile conditions.

### Intranasal infection.

Bats and mice were anesthetized by intramuscularly injecting a mixture of ketamine (100 mg/kg)/xylazine (10 mg/kg) (Mexican Official Guide, NOM-062-ZOO-1999) to ease the intranasal infection. In every assay, 12 animals from each species were infected with the standardized inoculum, dispensing 20 μL of the inoculum into each animal’s nostrils at intervals of approximately 30 seconds, until completing 100 μL.

High inoculum was used to ensure that many infective propagules were introduced into each animal to easily detect the fungal dimorphic transition and to facilitate the inoculum’s passage across the mucosa of the upper respiratory tract.

Bats and mice were euthanized appropriately, as recommended by UNAM’s Animal Care and Use Committee, at 10, 20, and 40 minutes and at 1, 2, and 3 hours postinfection, using two animals from each species every infection time. Two noninfected bats and mice were used as negative controls. The infection assay was processed in duplicate. Dissected noses (with NALT and NM) and CLN fragments were harvested from all animals, under sterile conditions in a biosafety cabinet. Each tissue sample was washed three times in sterile SS before the subsequent assays.

### Fungal recovery from infected animals and identification of their DNA polymorphic patterns.

All noses and CLN fragments were incubated on mycobiotic agar (Bioxon) for 3–4 weeks at 28°C to facilitate fungal growth and identification.^18^ The genomic DNA from each recovered fungal colony was evaluated by randomly amplified polymorphic DNA–polymerase chain reaction (RAPD-PCR) to confirm the polymorphic DNA pattern of *H. capsulatum*, as described by Taylor et al*.*^19^

### Paraffin-embedded sections.

Nose and CLN samples taken from each animal at the different postinfection times were fixed with 4% paraformaldehyde in 0.1 M Tris-HCl buffer (pH 7.2) for 8 hours. The nasal cavity and its bony framework, including the nasopharyngeal duct, were dissected after removing the soft tissues and bones from the animal’s head. The resulting bony block was decalcified by immersion in 10% ethylenediaminetetraacetic acid prepared in 0.1 M phosphate-buffered saline (pH 7.2) for 2 weeks. All specimens were paraffin embedded to obtain serial coronal nasal sections (containing NALT and NM) and longitudinal CLN tissue sections. All tissue sections were processed for periodic acid–Schiff (PAS), hematoxylin–eosin (H&E), and immunohistochemistry (IHC) stains.

### Detection of *H. capsulatum* and DCs in NM and CLN sections by IHC, using an immunoperoxidase stain.

Tissue sections (3 m) were mounted on positively charged Superfrost plus^®^ slides (Shandon Inc., Pittsburgh, PA) and processed according to the standardized immunoperoxidase method to detect *H. capsulatum* as described by Espinosa-Avilés et al*.*^20^ and Pérez-Torres et al.,^21^ using 3-amino-9-ethylcarbazole as the chromogen.

A second immunoperoxidase stain was performed to identify the DEC-205–positive DCs, using a rat anti-DEC-205 monoclonal antibody (NLDC-145; Serotec Co., Oxford, UK). A biotinylated goat anti-rat IgG (Zymed Laboratories Inc., San Francisco, CA) was used as the secondary antibody. The streptavidin–biotin–horseradish peroxidase complex was processed as described by Espinosa-Avilés et al*.*^20^ and Pérez-Torres et al.,^21^ using 3,3′-diaminobenzidine (Zymed) as the chromogen.

All the IHC slides were counterstained with Mayer’s hematoxylin for 30 seconds and mounted with hydrosoluble resin (Biocare Medical Co., Concord, CA).

Sections of a peripancreatic lymph node from a disseminated histoplasmosis clinical case and sections of a mouse thymus containing DEC-205–positive interdigitating DCs were used as positive controls in all IHC assays. Rabbit or rat normal sera were used as negative controls.

Photomicrographs were taken with a BX 50 microscope equipped with a digital camera (Olympus American Inc., Miami, FL).

### *Histoplasma capsulatum* phase–specific gene expression by reverse transcription–polymerase chain reaction (RT-PCR).

First, to standardize the optimal conditions for expressing *MS8*^10^ and YPS3^22^ phase–specific genes, we used suitable cultures of either the M- or Y-morphotype of the *H. capsulatum* EH-53 strain. The M-phase was grown at 28°C in BHI agar (Bioxon); the Y-phase was grown at 37°C in BHI broth (Bioxon) supplemented with 0.1% L-cysteine and 1% glucose. The RT-PCR assays were processed using total RNA extracted from each harvested morphotype, using TRIzol^®^ reagent (Invitrogen Life Technologies, San Diego, CA) as next described, both for NM and CLN samples.

Total RNA from previously deparaffinized tissue sections of each infected animal, at the different postinfection times studied, was extracted with TRIzol (Invitrogen). Briefly, 1 mL of TRIzol was added to 10 mg of NM or CLN deparaffinized samples, and each suspension was incubated at room temperature for 20 minutes, vortexing every 5 minutes. Next, 200 μL of chloroform was gently mixed at room temperature for 3 minutes. Each sample was centrifuged at 12,000 × *g* for 15 minutes at 4°C, the water-soluble phase was recovered, and 500 μL of isopropanol was added. Each sample was incubated at room temperature for 10 minutes and centrifuged at 12,000 × *g* for 10 minutes at 4°C. The supernatant was removed, and each pellet was suspended in 75% ethanol. Each pellet was again centrifuged at 7,500 × *g* for 5 minutes at 4°C and dried, dissolved in Milli-Q water, and, finally, stored at –80°C for further cDNA synthesis.

We designed two primer sets, with each set corresponding to a specific fungal morphotype: the M-phase MS8-primers, MS8-FWD (5′-GGGTTCTTCGAACTTCCTTG-3′) and MS8-REV (5′-TGAAGATATGCGGTACAACA-3′), which generated a 153-bp fragment of the *MS8* gene, and the Y-phase YPS3-primers, YPS-FWD (5′-TCTGCGGCACCTGCAACCCTAT-3′) and YPS-REV (5′-CGGCTTCGTGTTATCGTCGC-3′), which generated a 230-bp fragment of the *YPS3* gene.

The *MS8* gene encodes a predicted 21-kDa structural protein necessary for forming the hyphal cell wall.^11^ The *YPS3* gene encodes a 20-kDa protein localized in the yeast cell wall.^23–25^

All purified RNA samples (1 μg) were fluorometrically quantified and immediately processed for cDNA synthesis and amplification of each *H. capsulatum* phase–specific gene with the SuperScript III One-Step RT-PCR System (Invitrogen), using each primer set (10 pmol/primer) for *MS8* or *YPS3* genes. The following thermocycling conditions were used for cDNA synthesis and amplification: one cycle at 50°C for 30 minutes and one cycle at 94°C for 2 minutes, followed by 35 cycles at 94°C for 15 seconds, 55°C for 30 seconds, 72°C for 1 minute, and a final extension cycle at 72°C for 7 minutes.

Milli-Q water and/or RNA from the NM or the CLN of noninfected animals treated by the One-Step RT-PCR System were used as negative controls.

In all RT-PCR assays using RNA extracted from animal tissues, β-actin was processed as endogenous NM and CLN control samples for each infected and noninfected animal tested, using the primers (10 pmol each) FWD (5′-CCAACTGGGACGACATGG-3′) and REV (5′-GGTGGTACCACCAGACAGC-3′), which generated a 648-bp fragment. RT-PCR assays were processed in duplicate for each group of animals, according to each studied time.

The amplified products of all RT-PCT assays were electrophoresed on 1.5% agarose in 1× TAE buffer at 100 V for 50 minutes. A 123-bp DNA ladder (Gibco, Life Technologies, Carlsbad, CA) was used as a molecular size marker. The amplified bands were visualized with an ultraviolet transilluminator after staining with 0.5 g/mL ethidium bromide, and images were captured as TIFF files.

## RESULTS

### Fungal recovery from infected animals and identification of their DNA polymorphic patterns.

Three *H. capsulatum* mycelial colonies were recovered from the CLN of two bats at 2 and 3 hours postinfection and from the CLN of one mouse at 3 hours postinfection. *Histoplasma capsulatum* was identified by its typical macro- and micromorphology. Each recovered fungal colony was confirmed to be the same EH-53 strain from the original inoculum by comparing their RAPD-PCR polymorphic patterns (data not shown).

### Histological findings.

**Figure 1.**
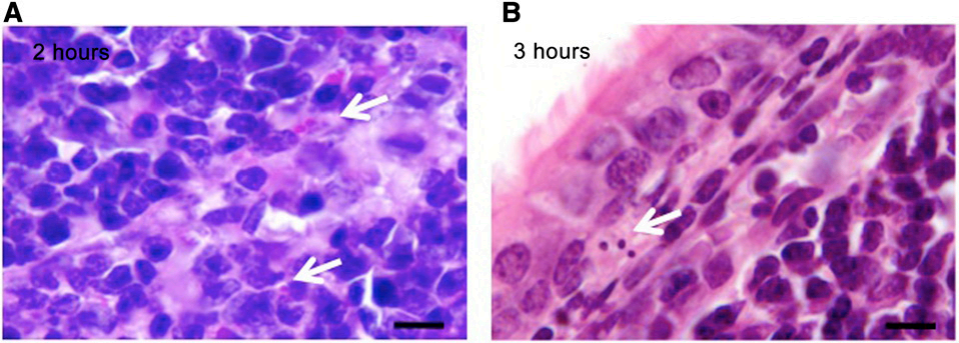
*Histoplasma capsulatum* yeast cells in the NALT of bats and mice after infection with mycelial propagules. (**A**) In bat NALT at 2 hours, periodic acid–Schiff stain. (**B**) In mouse NALT at 3 hours, hematoxylin–eosin stain. Arrows indicate yeast-like cells. Bars = 10 μm. NALT = nasal-associated lymphoid tissue. This figure appears in color at www.ajtmh.org.

Nasal-associated lymphoid tissue from both bats and mice was identified using transversal decalcified nasopharynx sections stained by PAS and H&E, and scarce free fungal cells compatible with *H. capsulatum* yeasts were observed in the subepithelial connective tissue and within NALT phagocytes in bats, at 2 hours postinfection, and in mice, at 3 hours postinfection (Figure 1A and B).

**Figure 2.**
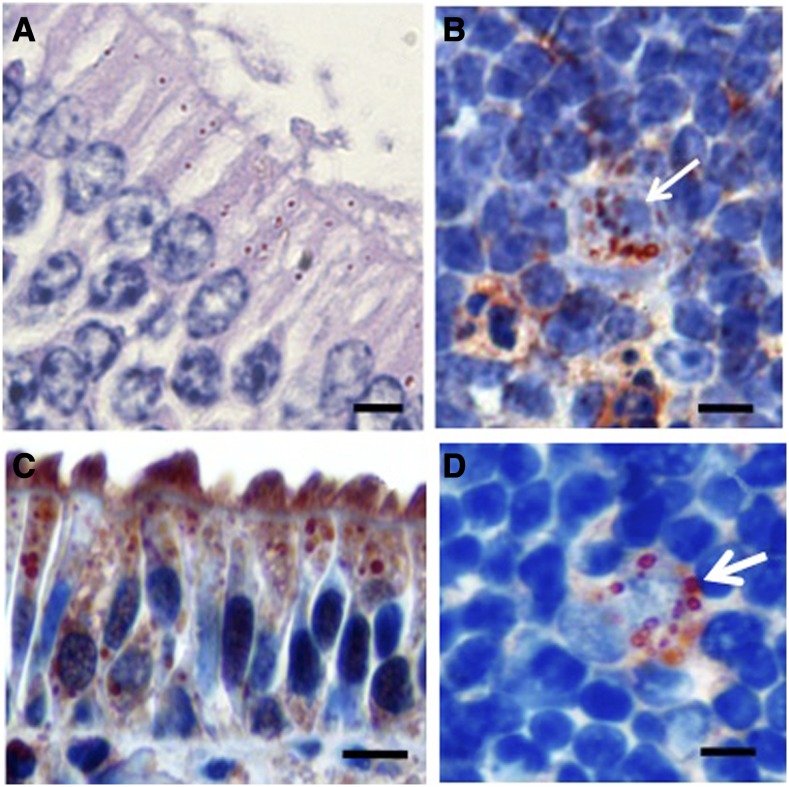
*Histoplasma capsulatum* yeast cells in the NM and the CLN of bats and mice at 3 hours postinfection with mycelial propagules. (**A**) In bat NM epithelial cells. (**B**) In mouse NM epithelial cells. (**C**) Within bat CLN phagocytes. (**D**) Within mouse CLN paracortical DC. All tissue sections were IHC stained: the yeast-like cells were observed in red color using 3-amino-9-ethylcarbazole as the chromogen, whereas Dendritic cells were observed in brown color using 3,3′-diaminobenzidine as the chromogen. The IHC sections were counterstained with Mayer’s hematoxylin. The selected NM sections did not enclose the nasal-associated lymphoid tissue. Arrows indicate yeast-like cells. Bars = 20 μm. CLN = cervical lymph nodes; IHC = immunohistochemistry; NM = nasal mucosa. This figure appears in color at www.ajtmh.org.

*Histoplasma capsulatum* yeasts were observed by IHC in the NM nonassociated to the NALT, in bats at 2 and 3 hours postinfection (Figure 2A) and in mice at 3 hours postinfection (Figure 2B). In general, fewer yeast cells were observed in bats’ NM epithelial cells than in those of mice. In most cases, yeasts were observed intracellularly, although they were occasionally found extracellularly. Likewise, intracellular and/or extracellular yeasts were observed by IHC in the bats’ CLN section at 2 and 3 hours postinfection, and in mice, at 3 hours postinfection (Figure 2C). Several yeast cells were found in the cytoplasm of phagocytes located in the paracortical zone of the CLN, mostly corresponding to interdigitating DCs (Figure 2D); also, the double IHC stain for *H. capsulatum* and DCs supported yeast phagocytosis by DCs (Figure 2D).

Numerous intracellular and extracellular yeasts were observed in peripancreatic lymph node sections from a clinical case of a fatal histoplasmosis that was used as a positive control for *H. capsulatum* infection. Immunohistochemistry of the thymus sections confirmed the assay’s usefulness for detecting DCs in tissues. In all IHC assays, controls with normal serum were always negative.

### Detection of *H. capsulatum* phase–specific gene expression by RT-PCR.

Expression of each morphotype-specific gene was standardized using RNA from each *H. capsulatum* morphotype culture. Total RNA concentrations determined for the M- and Y-phase cultures were 194 µg/mL and 228 µg/mL, respectively. After resolving the cDNA products on agarose gel electrophoresis, the *H. capsulatum* morphotypes were confirmed by the observation of the corresponding 153-bp (*MS8* gene) and 230-bp (*YPS3* gene) cDNA bands of the M- and Y-phase–specific genes, respectively (see Supplemental Figure 1).

**Figure 3.**
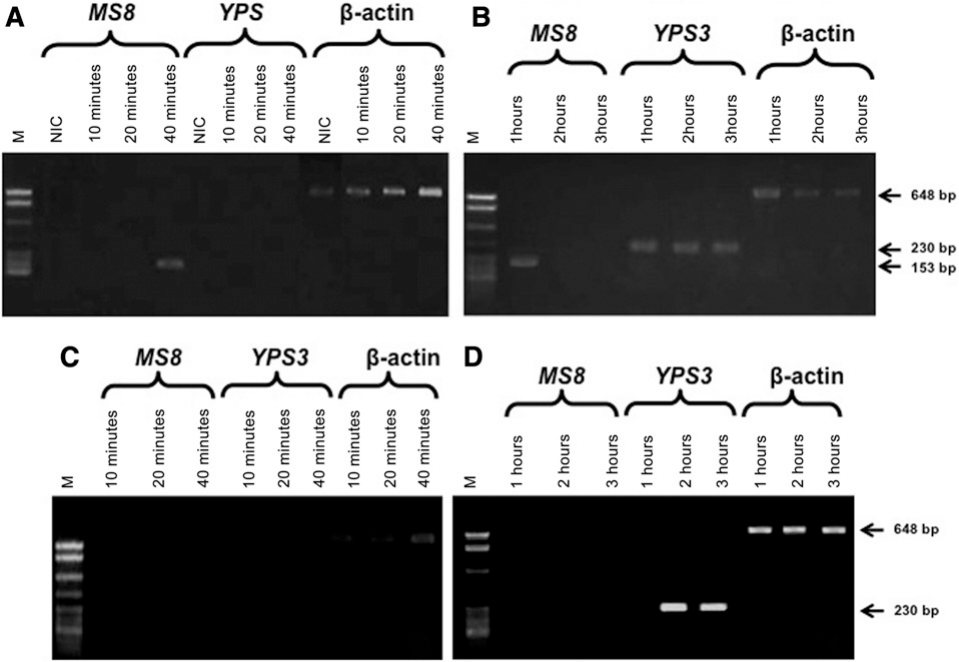
Representative expression of the *MS8-* and *YPS3*-phase–specific genes of *Histoplasma capsulatum* in the NM and the CLN of infected bats, at different times after infection. RT-PCR was used to identify the *H. capsulatum* phase–specific genes in each tissue sample, and the resulting cDNAs were resolved on agarose gel by electrophoresis (details are provided in the Materials and Methods section). Gel electrophoresis of bats’ NM: (**A**) from 10 to 40 minutes and (**B**) from 1 to 3 hours. Gel electrophoresis of bats’ CLN: (**C**) from 10 to 40 minutes and (**D**) from 1 to 3 hours. M: molecular marker (123-bp DNA ladder); NIC. Nasal mucosa or CLN β-actin cDNAs were used as an endogenous control for RT-PCR assays. Arrows indicate the amplified products of each RT-PCR reaction, corresponding to the cDNA for the *MS8* (153-bp), *YPS3* (230-bp), and β-actin (648-bp) genes. CLN = cervical lymph nodes; NM = nasal mucosa; NIC = noninfected control; RT-PCR = reverse transcription-polymerase chain reaction.

The RT-PCR conducted with the NM from infected bats revealed that the cDNA band matching the *MS8* gene was detectable from 40 minutes to 1 hour after infection (Figure 3A), whereas the band corresponding to the *YPS3* gene was revealed at 1 hour postinfection, remaining detectable up to 3 hours postinfection (Figure 3B). Conversely, in the bats’ CLN, the *MS8* gene expression was never detected at the postinfection times assayed (Figure 3C and D).

**Figure 4.**
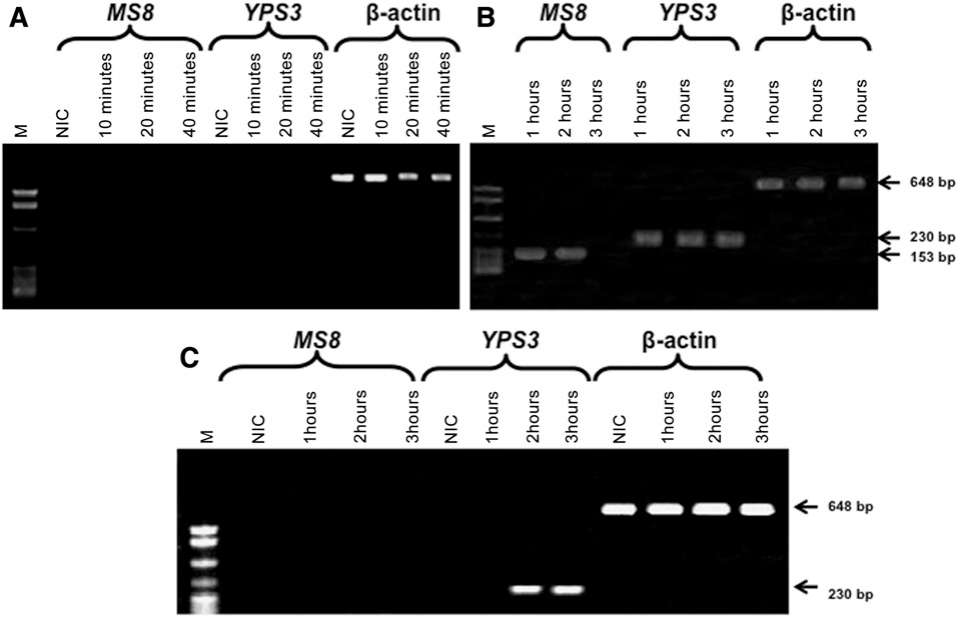
Representative expression of the *MS8-* and *YPS3*-phase–specific genes of *Histoplasma capsulatum* in the NM and the CLN of infected mice at different times after infection. RT-PCR was used to identify the *H. capsulatum* phase–specific genes in each tissue sample, and the resulting cDNAs were resolved on agarose gel electrophoresis (details are provided in the Materials and Methods section). Gel electrophoresis of mice NM: (**A**) from 10 to 40 minutes and (**B**) from 1 to 3 hours. Gel electrophoresis of mice CLN: (**C**) from 1 to 3 hours postinfection. M: molecular marker (123-bp DNA ladder); NIC. Nasal mucosa or CLN β-actin cDNAs were used as an endogenous control for RT-PCR assays. Arrows indicate the amplified products of each RT-PCR reaction, corresponding to the cDNA for the *MS8* (153-bp), *YPS3* (230-bp), and β-actin (648-bp) genes. CLN = cervical lymph nodes; NM = nasal mucosa; NIC = noninfected control; RT-PCR = reverse transcription-polymerase chain reaction.

In the mice NM, the band matching the *MS8* cDNA was not demonstrated during the initial infection stage (Figure 4A), but it was revealed from 1 to 2 hours postinfection (Figure 4B), whereas the band corresponding to the *YPS3* cDNA was found 1 hour postinfection and it remained evident during the subsequent assayed postinfection times (Figure 4B). Regarding *MS8* and *YPS3* transcript expression in the mice CLN, the 153-bp band corresponding to the *MS8* cDNA was never detected at any of the postinfection times assayed (Figure 4C), whereas the 230-bp band corresponding to the *YPS3* cDNA was demonstrated at 2 and 3 hours postinfection (Figure 4C).

The 648-bp band corresponding to the β-actin cDNA (tissue endogenous control) was revealed in all assays.

The RT-PCR assay performed with Milli-Q water and RNA from the tissues of noninfected animals systematically showed negative results.

## DISCUSSION

The interaction between *H. capsulatum* morphotypes and host responses associated with the upper respiratory tract was never explored before. The NM represents the host’s first defense site against the entry of airborne pathogens into the upper and lower respiratory tracts. In humans, the upper respiratory airways, particularly the highly vascular NM, have important protective functions, which include humidifying and heating of inspired air to 37°C.^26^ Thus, human hosts’ physical characteristics could induce a fungal M-to-Y–phase switch, which is crucial for fungal virulence and survival in hostile intra- and extracellular host environments.

In this study, remarkable molecular approaches were used to demonstrate *H. capsulatum* dimorphic transition and its subsequent dissemination after intranasal infection with a mycelial inoculum in bats and mice, to determine the roles played by the NM, NALT, and CLN of both animal models, in response to the challenge yielded by a fungal pathogen primarily associated with the respiratory tract. In mice, the NALT is well known,^27,28^ whereas the NALT of bats was described for the first time by Suárez-Alvarez et al.^29^ Although, there are slight anatomic differences in the NALT of both animals, histologically, they share the same lymphoid tissue organization.^29^

Here, we observed by IHC that *H. capsulatum* yeasts were present within the epithelial cells and the subepithelial DCs of the NM, as well as within the paracortical DCs of the CLN, in both bats and mice. Our findings suggest differences between the two animal models, regarding the number of fungal cells within the host’s epithelial cells, the time required for the dimorphic transition and the subsequent fungal dissemination to the CLN, which could induce slight discrepancies in their immune responses to histoplasmosis infection. Overall, low inflammatory responses were reported in the lungs of infected bats, which showed histological and fungal culture findings compatible with *H. capsulatum* as described by Taylor et al.^18^ Thus, bats can resolve the *H. capsulatum* infection through a more effective response at first contact with the fungus, which may differ from the mice defense mechanisms.

Although the *H. capsulatum* M-to-Y transition and further dissemination are classically associated to alveolar macrophages,^2^ the present study showed that the NM may also be involved in both processes. Therefore, we suggest that the time elapsed during the *H. capsulatum* morphotype transition in infected hosts may be vital for fungal survival after morphotype transition and dissemination, and that it could differ drastically from the time expected under nonnatural conditions. In addition, several mechanisms related to the innate immune response of the *H. capsulatum* infection route are implicated in the host–fungal interaction, and they occur simultaneously in different respiratory anatomic sites of the infected host. Sahaza et al.^17^ suggested that the time spent in the in vivo dimorphic transition modulates the cytokine production and granuloma development, during the pulmonary response of male BALB/c mice that were intranasally infected with M-phase propagules. Besides, recovering cultured *H. capsulatum* from the CLN of both animal models at 2–3 hours postinfection demonstrated a fast infectious process and it also confirmed the inocula viability and the occurrence of fungal dissemination to the CLN in bats and mice.

Here, we stated that in vivo fungal dimorphic transition occurred in a shorter time than the time recorded in different studies reporting dimorphism in vitro^30–32^ or using mice infection with *H. capsulatum* conidia.^33,34^ Notably, we monitored the fungal presence preferentially in the upper respiratory sites after short periods of time postinfection, using morphotype-specific molecular markers of *H. capsulatum*. In addition, yeast presence in the CLN at 2–3 hours as demonstrated by RT-PCR, with no data on the M-morphotype, provides unquestionable evidence of early fungal dissemination.

The RT-PCR system selected for the present study was an excellent tool for distinguishing the M- or Y-phase of *H. capsulatum* because it discriminated between the expressions of each morphotype-specific gene in the infected tissue samples; also, the use of endogenous β-actin processed as tissue control in different assays indicated this methodology’s accurate development. Besides, the restricted expression of the *MS8* and *YPS3* genes by RT-PCR assays, only in the corresponding *H. capsulatum* M- or Y-phase cultures, confirmed that both markers were morphotype specific.

RT-PCR analyses also demonstrated that the phase-specific *MS8* and *YPS3* genes were expressed in infected bats and mice, albeit with some differences in their time-points (see Figures 3 and 4). Our findings suggest that the dimorphic transition of *H. capsulatum* occurs earlier in bats than in mice and that the first 2 hours after infection could be proposed as a necessary time for the in vivo fungal morphotype transition process, which was completed at 3 hours. The authors acknowledge that intrinsic conditions of both hosts (bats and mice) could differentially influence the *H. capsulatum* transition process, as some discrepancies were observed along the infectious course in these animal models.

Interestingly, the *MS8* gene was no longer expressed in the NM in either bats or mice at the latest infection time analyzed, suggesting that all mycelial propagules that crossed the NM were converted to Y-morphotype before arriving to the CLN. Identification of only the Y-phase in the CLN suggests that phagocytes associated with the NM and the NALT, usually the DCs, transport the fungus through the blood and lymphatic vessels, draining these tissues, and carrying the fungus toward the CLN. It is also possible that some extra- or intracellular yeast cells, non-processed in the CLN, could continue to be transported by hepatic and/or lymphatic routes, thus facilitating the Y-morphotype dissemination to other host tissues. Our findings suggest that DCs are ideal candidates for mobilizing the fungus from the infected animals’ NM to their CLN. Identification of intracellular yeast-like cells within the subepithelial and interstitial DCs of the NM and within the interdigitating DCs of the CLN in both animal models also supports this statement. Although other strategies are involved in disseminating *H. capsulatum*,^35,36^ a DC-based mechanism appears to be the easiest route for this pathogen to arrive to the CLN.

Given the participation of the NM and the NALT described in this study, it is relevant to emphasize that these tissues have all the elements required to mount a competent local immune response. The efficacy of this defense depends on diverse factors, including the fungal inoculum, the *H. capsulatum* phylogenetic species and its virulence, the mammalian species infected, and the host’s whole immunological state.

The present findings do not exclude the classic route involved in *H. capsulatum* infection, in which aerosolized infective M-phase propagules follow a direct route, arriving to the lungs, where the dimorphic transition and dissemination are expected to occur.^2,3^ However, we demonstrated that fungal dimorphism and initial dissemination in infected animals might also occur in the NM without fungal propagules having to arrive to the lungs.

Finally, it is important to mention that our indigenous EH-53 *H. capsulatum* strain, which was previously classified as a LAm A phylogenetic species, has expressed the *YPS3* transcript either in the fungal culture or in the infected tissue, as detected by RT-PCR assays in the present study. These findings are relevant because neither the *YPS3* transcript nor its protein has been previously identified in the *H. capsulatum* LAm A phylogenetic species.

## CONCLUSION

The results of the morphological, immunohistochemical, and molecular assays discussed so far, which were performed on the tissues of infected bats and mice, first suggest that in the infected host, *H. capsulatum* has alternative sites for initiating its dimorphic transition and early dissemination, including the NM, the NALT, and the CLN, and, second, that the time required for *H. capsulatum* dimorphic transition under in vivo infection conditions, mimicking the natural route of pulmonary histoplasmosis, was shorter than the time reported by other studies performed under nonnatural conditions, such as using non-appropriated hosts, noninfective *H. capsulatum* propagules, and an inaccurate route of fungal infection.

## Supplementary Files

Supplemental figure
