# Supplementary material for: Dimorphism and Dissemination of Histoplasma capsulatum in the Upper Respiratory Tract after Intranasal Infection of Bats and Mice with Mycelial Propagules
Source: Am J Trop Med Hyg. 2019 Jul 8;101(3):716–23. doi: 10.4269/ajtmh.18-0788 (PMC6726946; doi:10.4269/ajtmh.18-0788)

Supplementary Figure 1. Expression of the *MS8* and *YPS3* phase-specific genes of *H. capsulatum* from mycelial and yeast cultures in suitable media. RNA extracted from each M- and Y-phases of *H. capsulatum* EH-53 strain cultures were processed by RT-PCR and the resulting cDNAs were resolved on agarose gel electrophoresis (details are provided in the Materials and Methods section). M, molecular marker (123-bp DNA ladder); MS8, M-phase culture (153 bp); YPS3, Y-phase culture (230 bp); C (-) negative control.

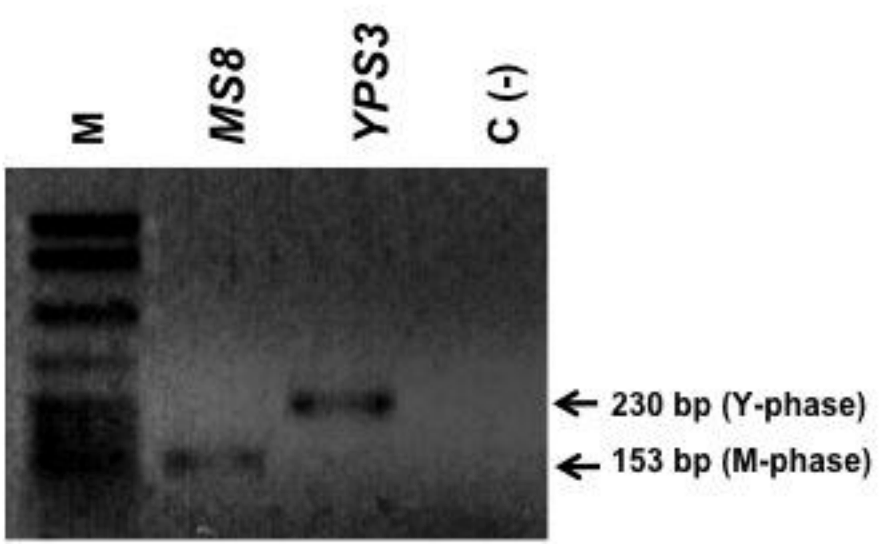

Supplement: Supplementary file 1 [file tpmd180788.SD1.pdf]
